# Effect of PCSK9 Inhibitors on Hemostasis in Patients with Isolated Hypercholesterolemia

**DOI:** 10.3390/jcm11092542

**Published:** 2022-05-01

**Authors:** Marcin Basiak, Marcin Hachula, Michal Kosowski, Boguslaw Okopien

**Affiliations:** Department of Internal Medicine and Clinical Pharmacology, Medical University of Silesia, Medyków 18, 40-752 Katowice, Poland; marcin.hachula@gmail.com (M.H.); mkosowski@sum.edu.pl (M.K.); bokopien@sum.edu.pl (B.O.)

**Keywords:** PCSK9 inhibitor, factor VII, hypercholesterolemia, plasminogen activator inhibitor-1, von Willebrand factor, hemostasis, hyperlipidemia, fibrinogen, new lipid-lowering drugs

## Abstract

Background: In addition to reducing plasma lipids, proprotein convertase subtilisin/kexin type 9 (PCSK9) inhibitors may produce numerous nonlipid-related pleiotropic effects. The purpose of this trial was to determine the efficacy of PCSK9 inhibitors alone in patients with isolated hypercholesterolemia. Methods: The trial enrolled 21 individuals with isolated hypercholesterolemia and atherosclerosis who received alirocumab for 90 days (150 mg every two weeks). Lipids, glucose homeostasis factors, and hemostatic markers were measured in the plasma at baseline and after treatment. Results: The PCSK9 inhibitor administered to these patients reduced plasma levels/activity of fibrinogen (from 3.6 ± 0.5 to 2.9 ± 0.4 g/L, *p* < 0.01), factor VII (from 143.8 ± 16.7 to 114.5 ± 14.1%, *p* < 0.01) and plasminogen activator inhibitor-1 (PAI-1) (from 74.9 ± 13.9 to 52.8 ± 9.1 ng/mL, *p* < 0.001) without a significant reduction in von Willebrand factor levels, and it tended to prolong the partial thromboplastin and prothrombin times. Conclusion: Our findings indicate that treatment with PCSK9 inhibitors has a multipotential effect on fibrinolysis and coagulation in patients with isolated hypercholesterolemia and that this medication may have some future benefits in patients who are statin-intolerant or contraindicated to statin use.

## 1. Introduction

Worldwide, atherosclerosis and its consequences are the primary causes of morbidity and mortality, accounting for more than 19 million deaths per year [1]. Previous research has demonstrated that impaired activity of fibrinolysis and coagulation contributes to the development and progression of atherosclerosis as well as to the occurrence of atherosclerotic cardiovascular events [2,3]. Because fibrinolysis and coagulation abnormalities may be implicated in the early and late stages of this process and its repercussions, normalizing them may protect the patient from atherosclerosis progression and the development of acute vascular incidents [4]. VLDL, LDL, and oxLDL all contribute to thrombus formation, but HDL has antithrombotic effects [5,6]. PCSK9 inhibitors are a class of drugs that work by binding to the PCSK9 molecule and preventing it from attaching to the low-density lipoprotein cholesterol receptors (LDL-Rs), hence inhibiting its degradation, increasing the absorption of the low-density lipoprotein cholesterol (LDL-C) from the bloodstream, and lowering its concentration [7]. Recently, the antiplatelet effects of PCSK9 inhibitors were thoroughly reviewed [8]. Data from experimental studies in animals suggest that PCSK9 can modulate both primary and secondary hemostasis indirectly, by affecting LDL-C, or directly, by affecting platelet activation and plasma factor VIII levels [9]. Prothrombin time (PT) and activated partial thromboplastin time (APTT) are involved in the internal and external coagulation pathways, respectively, which are associated not only with venous but also arterial thrombosis [10,11].

Despite the fact that PCSK9 inhibitors have a similar quantitative effect on LDL cholesterol reduction as statins, there are no clear findings on their effect on hemostatic markers at this time. More importantly, it is believed that PCSK9, in addition to regulating the level of LDL-C in the plasma, is associated with procoagulation, enhancing the development of atherosclerosis [4]. Previous studies showed that the concentration of circulating PCSK9 was independently associated with fibrinogen, the main protein associated with circulating blood coagulation, in patients with stable coronary artery disease (CAD) [12]. Importantly, to the best of our knowledge, only a few previous studies have examined whether PCSK9 inhibitors induce any changes in hemostasis in patients with hypercholesterolemia. This study aimed to investigate whether PCSK9 inhibitors had any effect on the markers of coagulation and fibrinolysis in subjects with primary isolated hypercholesterolemia.

## 2. Materials and Methods

Patients aged 18–75 years old were enrolled in the study, which was a small, short-term, non-randomized, and controlled study. They were evaluated in our department for the existence of asymptomatic atherosclerosis using sonographic measurement of the common carotid intima-media thickness. The study was conducted between the end of 2019 and 2021. Patients were eligible for the study if they met the following criteria: isolated hypercholesterolemia (former Frederickson hyperlipidemia type 2A)—total cholesterol > 200 mg/dL, LDL-cholesterol > 135 mg/dL, triglycerides < 150 mg/dL; ineffective dietary treatment for at least 3 months; common carotid intima–media thickness > 1.0 mm; for women, at least 24 months since last menstruation, ovariectomy, or hysterectomy, or use of highly effective mechanical contraception.

The exclusion criteria were as follows: primary mixed dyslipidemia or hypertriglyceridemia; secondary dyslipidemia in the course of autoimmune disorders, thyroid diseases, chronic pancreatitis, nephrotic syndrome, liver and biliary tract diseases, obesity (BMI > 30 kg/m^2^) or alcoholism; symptomatic congestive heart failure; unstable coronary artery disease, myocardial infarct or stroke in the 6 months preceding the study; arterial hypertension; impaired renal or hepatic function; malabsorption syndromes; any acute or chronic inflammatory processes; treatment with other hypolipidemic drugs in the 3 months before the study; taking drugs interfering with studied substances’ serum levels or affecting lipid metabolism (e.g., statins, fibrates, ezetimibe, niacin, and non-selective beta-blockers); concomitant treatment with drugs that may affect inflammatory processes in the vascular wall (including non-steroid anti-inflammatory drugs and angiotensin-converting enzyme inhibitors) in the 3 months before enrollment; and ongoing hormonal replacement therapy or oral contraception.

**Figure 1:**
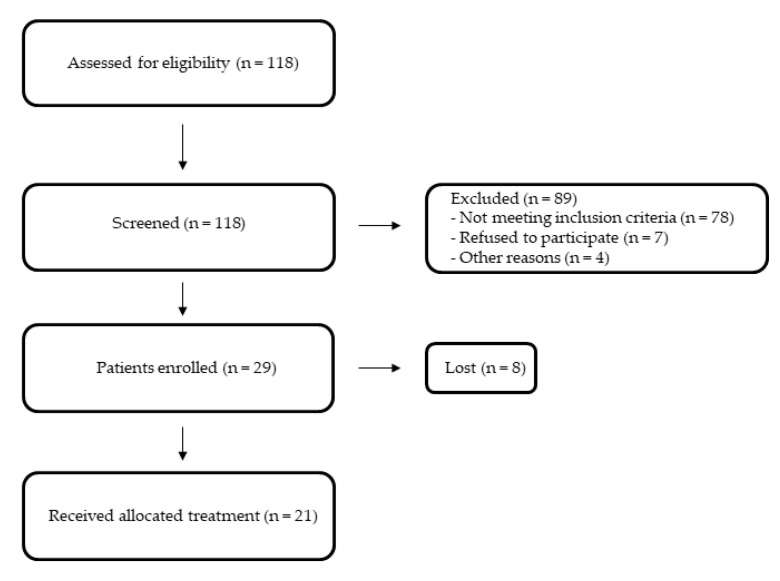
Study flow chart.

Of the 118 patients screened, 21 were eligible for study entry and met all entry criteria (Figure 1).

Each patient provided written informed consent in accordance with the Helsinki Declaration. The Medical University of Silesia’s Bioethical Committee accepted the study protocol. Patients were placed on a low-fat diet for at least three months prior to and during the research. Because lipid-lowering medicine has been demonstrated to be beneficial, the use of a placebo group was deemed unethical. As a result, our control group consisted of 19 healthy volunteers who were age-, sex-, and weight-matched. Before and 90 days after the initiation of therapy, a history was taken, a clinical examination was performed, and venous blood was drawn to evaluate safety laboratory data. Constipation, dyspepsia, abdominal pain, flatulence, and myalgia were all carefully questioned in these patients. Blood pressure was measured on both upper limbs, and the one with the higher result was chosen. Then the RR was measured three times (automatic measurement with a break of at least 2 min in a sitting position after at least 5 min of rest). The values were averaged. Laboratory tests included total and differential blood cell count, blood sedimentation rate, creatine kinase, alanine and aspartate aminotransferases, alkaline phosphatase, gammaglutamyltransferase, electrolytes, creatinine, bilirubin, total proteins, urine examination, glycated hemoglobin (at the beginning and end of the study), and 12-lead ECG. Safety parameters did not differ between study groups.

All included patients who complied throughout the entire study period with lifestyle modifications were treated with a constant dose of alirocumab (150 mg), administered every two weeks at the same time of the day for 90 days.

Venous blood samples were collected after an overnight 12 h fasting at 8 a.m. before and after 90 days of PCSK9 inhibitor treatment. All tests were performed by a clinical analyst who did not know the patient’s identity or any clinical details. Plasma lipids and glucose were assayed by routine laboratory techniques (Beckman, Palo Alto, CA, USA; bioMérieux, Marcy l’Etoile, France; Linco Research Inc., St. Charles, MO, USA; Bayer Ames Technicon, Tarrytown, NY, USA); LDL cholesterol levels were measured directly. Prothrombin and partial thromboplastin times, factor VII coagulant activity, fibrinogen, plasminogen activator inhibitors type 1 (PAI-1), and von Willebrand factor (vWF) were assessed. Prothrombin and partial thromboplastin times were determined by an automated blood coagulation analyzer (Sysmex CA-540 and Dade Behring Marburg, Germany, reagents). Fibrinogen and factor VII were determined by a semi-automated blood coagulation analyzer (Option-2 Plus and bioMérieux reagents Marcy l’Etoile, France). PAI-1, andvWF levels were determined by commercially available ELISA methods (Asserachrom, Diagnostica Stago, France). All laboratory tests were also performed in the control group at baseline and after 90 days.

### Statistics

The collected data were processed using the Statistica TIBCO Software Inc., Palo Alto, CA, USA (2017) version 13.3 program, licensed by the Medical University of Silesia in Katowice. We used the Kolmogorov–Smirnov test to assess the normality of distributions, which was confirmed by q-q plots. To fit a normal distribution curve, a log transformation was used for non-normal variables (hemostatic variables). To compare quantitative variables, the *t*-test for independent means and the *t*-test for dependent means were used. Student’s paired *t*-test was used to compare the means of variables within the same treatment group. For categorical variables, the χ^2^ test was used. We assumed a *p*-value of less than 0.05 to be statistically significant. Correlations were calculated using Kendall’s τ test.

## 3. Results

The study groups did not differ significantly in terms of demographic data (age, gender, smoking, and weight) (Table 1). After reviewing the patient records, it was discovered that patients in both groups had not received any medications in the three months preceding enrollment. All patients completed this study, and there were no significant adverse events or serious adverse events. Laboratory safety measurements were within normal limits. The control group remained without any effect on plasma lipids, the hemostatic variables measured, or glucose.

Alirocumab, on the other hand, decreased total and LDL cholesterol in the plasma, tended to decrease triglycerides (*p* = 0.086), and tended to enhance HDL cholesterol (*p* = 0.089). Additionally, the PCSK9 inhibitor reduced fibrinogen, factor VII activity, and PAI-1 levels, and it tended to reduce vWF (*p* = 0.062) (Table 2). Additionally, it tended to prolong prothrombin (*p* = 0.089) and partial thromboplastin (*p* = 0.072) times. With the exception of a slight association between triglycerides and PAI-1 (*r* = 0.42, *p* = 0.01) and triglycerides and factor VII (*r* = 0.44, *p* = 0.01), no correlations between baseline plasma lipids or glucose and baseline hemostatic indicators were observed. The effects of the PCSK9 inhibitor on coagulation and fibrinolysis were unrelated to the effect of the drug on plasma lipids or the measured glucose level (*r* values ranging from −0.10 to 0.26). After 90 days of treatment, there was no correlation between prothrombin and partial thromboplastin times, fibrinogen, factor VII, PAI-1, vWF, and plasma lipids.

## 4. Discussion

Our first results show that alirocumab used in patients with isolated hypercholesterolemia can inhibit key responses to stimulated fibrinolysis and the coagulation cascade. In this study, we used markers such as PAI-1, factor VII, fibrinogen, and vWF, for which high plasma levels or activities have been previously found to be associated with increased morbidity and mortality related to cardiovascular diseases [13,14].

The prolongation of the activated partial thromboplastin and prothrombin times, which indicate the activity of the intrinsic and extrinsic pathways of coagulation, respectively, were correlated with changes in the hemostatic factors we tested [15]. The effects of alirocumab on factor VII, PAI-1, and fibrinogen, along with the slightly smaller impact of PCSK9 inhibition on both clotting times, indicate an improvement in global hemostasis. Hemostatic disturbances are one of the most important factors in the development of atherosclerosis and its complications. Thus, the reduction in plasma levels and activity of the hemostatic variables examined as a result of PCSK9 inhibition may provide significant therapeutic benefits to hypercholesterolemic subjects taking this medication [16].

Recent studies have proven that PCSK9 promotes the development of atherosclerosis not only through the mechanism of increased plasma LDL-C concentrations but also through direct action on the cells that form the artery walls and atherosclerotic plaques. The FOURIER trial data indicated a significant reduction in LDL-C in the evolocumab-treated group. Similar results were reported in individuals enrolled in the ODYSSEY OUTCOMES trial, in whom plasma LDL-C levels were lowered by up to 62% when alirocumab was used versus when a placebo was used. They found that high efficacy in reducing cardiovascular risk does not appear to be entirely dependent on lipoprotein-lowering effects [17,18]. After multiple clinical trials demonstrated that combining lipid-lowering therapy with statins significantly reduced cardiovascular risk more than simply lowering plasma lipids, scientists began to examine alternative beneficial mechanisms underlying this occurrence. Similar findings regarding cardiovascular risk were obtained for PCSK9, which was shown to induce thrombosis and hypercoagulability [4]. Additional pleiotropic effects of PCSK9 inhibitors, including plaque stabilization, anti-atherosclerotic effects, antineoplastic effects, and the ability to impact the course of bacterial infections, have been comprehensively reviewed recently [19].

Importantly, PCSK9 directly and significantly reduces the level of circulating LDL-C by mediating lysosomal degradation of hepatic LDLR and exerts a strong influence on thrombosis processes [20]. Hence, the combination of PT and PCSK9 increases the incidence of cardiovascular incidents in patients with angina-like chest pain, which is beneficial for risk evaluation [20,21]. Our study suggests that therapy with PCSK9 inhibitors may be justified even in cases of mild hypercholesterolemia, especially if this treatment is used in patients with high cardiovascular risk.

Notably, PCSK9 inhibitors have an effect on the incidence of venous thromboembolism, which is associated with endothelial inflammation and atherogenesis. Alirocumab’s effects were unrelated to its effects on total, LDL, HDL, and triglyceride cholesterol. Thus far, no correlation has been established between LDL-C levels and the occurrence of venous thromboembolism. In the case of statins, hemostasis improvement is a side effect of the agent’s inhibitory effect on the mevalonate pathway, which is involved in the regulation of several critical steps in the fibrinolysis and coagulation processes [22]. Even during short-term treatment, fibrates have some extralipid effects on factor VII, fibrinogen, and type 1 plasminogen activator (PAI-1) [23]. It is more challenging to understand the mechanism underlying PCSK9 inhibition’s hemostatic effects. Statins have been employed in lipoprotein-lowering therapy to reduce LDL-C and triglyceride levels, but they have little effect on Lp(a) levels. It is critical to evaluate the plasma concentrations of LDL-C and Lp(a) when using PCSK9 inhibitors, which, in contrast to statins, reduce both [24]. As a result, clinical trials have been conducted to determine the effect of alirocumab on venous thromboembolism incidence. The results unequivocally verified alirocumab’s favorable effect on reducing the incidence of venous thromboembolism events, which was related to a considerable decrease in Lp(a) concentrations [25].

The second probable antithrombotic mechanism of PCSK9 inhibitor activity, which requires additional experimental research, is connected with their ability to improve the clearance of blood clotting factor VIII (FVIII), a critical protein in coagulation processes. Factor VIII is a plasma protein that is expressed by a gene on the X chromosome and is involved in clotting and coagulation. FVIII acts as a cofactor for FIX, thereby causing the production of thrombin. In our study, we also took into account von Willebrand factor, which transports FVIII, stabilizing it and reducing its clearance [26,27]. The hypothesis that PCSK9 inhibitors reduce the concentration of FVIII by increasing its clearance seems very feasible. The mechanism of PCSK9 inhibitors of increasing LDLR expression may increase the clearance of FVIII. As a result, the plasma level of FVIII is decreased, which may contribute to the reduction in the risk of cardiovascular events [28,29]. Our study has some limitations. The study did not assess clinical outcomes, such as the occurrence of cardiovascular events during prolonged follow-up. In addition, the study population size, although exceeding the required sample size, was relatively low. However, this is due to the fact that the number of patients treated with PCSK9 inhibitors in our country is rather low, and we choose very specific and difficult criteria for participants to meet. We also did not study important factors such as Lp(a), Apo B, or oxLDL because of the availability and budget during the COVID-19 pandemic, and these factors could show further pleiotropic effects of PCSK9 inhibitors or correlate with factors interfering with hemostasis. Diabetes belonged to the exclusion criteria, and the question of whether the PCSK9 inhibitor affects hemostasis in diabetic patients remains open.

## 5. Conclusions

Summing up, this small non-randomized study showed that PCSK9 inhibitors may affect hemostatic variables in subjects with isolated hypercholesterolemia. Numerous studies have shown that PCSK9 plays a significant role in cardiovascular disease, partially independent of its effect on lipid metabolism, and modern lipid-lowering therapy can help reduce cardiovascular risk in adults. Our findings indicate that PCSK9 may have an effect on primary and secondary hemostasis either indirectly, via its effect on LDL-C, or directly, via its effect on platelet activation and plasma FVIII levels; they also suggest that the therapy may be effective even in mild lipid disorders and in patients who are statin-intolerant or have contraindications to statin use. In actual clinical settings, PCSK9 inhibitors are very expensive, and so far, they are only recommended in selected patients with high or very high cardiovascular risk and those intolerant to statins. Because of numerous study limitations, the obtained results should be supported by a larger clinical trial.

## Figures and Tables

**Table 1:** Baseline characteristics of patients (values are mean ± SD unless indicated otherwise).

	Control Group	Study Group before Treatment
Number of patients	19	21
Age, years	45 ± 5	47 ± 6
Women, %	37	35
BMI	27.2 ± 2.7	28.1 ± 2.2
WHO guidelines on physical activity, %	84	81
Smokers, %	26	27
Alcohol abuse	No	No
Systolic blood pressure, mmHg	132 ± 6	134 ± 5
Diastolic blood pressure, mmHg	84 ± 4	83 ± 4
INR	0.84 ± 0.05	0.85 ± 0.06
Total cholesterol, mg/dL	160.2 ± 10.6	249.7 ± 12.9
LDL cholesterol, mg/dL	96.4 ± 8.7	181.0 ± 10.6
HDL cholesterol, mg/dL	48.1 ± 4.3	46.2 ± 4.2
Triglycerides, mg/dL	119.2 ± 9.6	121.6 ± 11.2
Fasting glucose, mg/dL	91 ± 4	92 ± 5
Prothrombin time, s	13.4 ± 1.3	14.2 ± 1.2
Partial thromboplastin time, s	31.1 ± 1.4	32.1 ± 1.7
Fibrinogen, g/L	3.2 ± 0.2	3.6 ± 0.5
Factor VII activity, %	141.3 ± 15.2	143.8 ± 16.7
Von Willebrand factor, IU/dL	116.1 ± 17.2	118.1 ± 16.0
PAI-1 antigen, ng/mL	72.3 ± 15.2	74.9 ± 13.9

**Table 2:** Effect of PCSK9-inhibitor on plasma lipids, glucose, fibrinolysis, and coagulation in patients with isolated hypercholesterolemia.

Variable		Control Group (Λ%)	Study Group (Λ%)
Total cholesterol, mg/dL	Baseline After 90 days	160.2 ± 10.6 157.1 ± 10.2 (−2)	249.7 ± 12.9 152.4 ± 10.2 (−39) ***^,###^
LDL cholesterol, mg/dL	Baseline After 90 days	96.4 ± 8.7 95.3 ± 8.1 (−1)	181.0 ± 10.6 108.3 ± 8.4 (−40) ***^,###^
HDL cholesterol, mg/dL	Baseline After 90 days	48.1 ± 4.3 49.1 ± 4.2 (2)	46.2 ± 4.2 48.2 ± 4.3 (4)
Triglycerides, mg/dL	Baseline After 90 days	119.2 ± 9.6 119.8 ± 10.2 (1)	121.6 ± 11.2 109.2 ± 9.4 (−10)
Fasting glucose, mg/dL	Baseline After 90 days	91 ± 4 92 ± 5 (1)	92 ± 5 90 ± 4 (−2)
Prothrombin time, s	Baseline After 90 days	13.4 ± 1.3 13.8 ± 1.4 (3)	14.2 ± 1.2 16.4 ± 0.7 (15)
Partial thromboplastin time, s	Baseline After 90 days	31.1 ± 1.4 31.8 ± 1.6 (2)	32.1 ± 1.7 36.6 ± 1.4 (14)
Fibrinogen, g/L	Baseline After 90 days	3.2 ± 0.2 3.5 ± 0.3 (9)	3.6 ± 0.5 2.9 ± 0.4 (−20) **^,#^
Factor VII activity, %	Baseline After 90 days	141.3 ± 15.2 142.2 ± 15.3 (1)	143.8 ± 16.7 114.5 ± 14.1 (−20) **^,##^
Von Willebrand factor, IU/dL	Baseline After 90 days	116.1 ± 17.2 120.2 ± 13.2 (4)	118.1 ± 16.0 96.8 ± 11.4 (−18) ^#^
PAI-1 antigen, ng/mL	Baseline After 90 days	72.3 ± 15.2 74.4 ± 12.8 (3)	74.9 ± 13.9 52.8 ± 9.1 (−30) ***^,###^

Values are mean ± SD unless indicated otherwise. ** *p* < 0.01, *** *p* < 0.001 versus baseline; ^#^ *p* < 0.05, ^##^ *p* < 0.01, ^###^ *p* < 0.001 versus control.

## Data Availability

The data that support the findings of this study are available from the corresponding author (mbasiak@sum.edu.pl) upon reasonable request.
